# Operation Limits of Integrated Photo‐Rechargeable Batteries

**DOI:** 10.1002/adma.74159

**Published:** 2026-07-17

**Authors:** Byung‐Man Kim, Arvind Pujari, Hooman Abbasi, Weidong Xu, Yutong Han, Samuel D. Stranks, Michael De Volder

**Affiliations:** ^1^ Institute for Manufacturing Department of Engineering University of Cambridge Cambridge UK; ^2^ Cavendish Laboratory Department of Physics University of Cambridge Cambridge UK; ^3^ Department of Chemical Engineering and Biotechnology University of Cambridge Cambridge UK

**Keywords:** light energy harvesting and storage, light‐induced charge transfer, photo‐charging current, photo‐rechargeable battery

## Abstract

Monolithic photo‐rechargeable batteries (PRB) are attractive solution for powering off‐grid autonomous systems. However, the fundamental effects limiting the light‐charging process in such devices are not well understood. Herein, we present an integrated PRB design that can be fully charged under a range of illuminances. We use it as a model system to correlate the decay in photo‐charging current with photo‐induced charge kinetics. Our results indicate that light‐induced hole transport gradually deteriorates with increasing state of charge, which is attributed to the anion‐coupled hole accumulation in the cathode layer. Furthermore, our device reveals that the potential gap between the hole transport layer and the cathode is critical for driving a photo‐induced delithiation of the cathode. If the cathode has a more positive delithiation potential than the hole transport level, the photo‐charging current rapidly decays despite the photo‐cells providing sufficient voltage to charge the battery. These findings demonstrate that the device physics of PRBs vary greatly from that of separately coupled solar cells and batteries, thus providing new insights in their working mechanism and their future design guidelines.

## Graphical abstract

This study elucidates the operational limitations of integrated photo‐rechargeable batteries. We demonstrate that the photo‐charging process is fundamentally restricted not only by material‐to‐material energy level alignment but also by transient capacitive charge accumulation within the cathode framework.

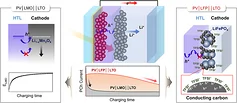

## 1 Introduction

**FIGURE 1:**
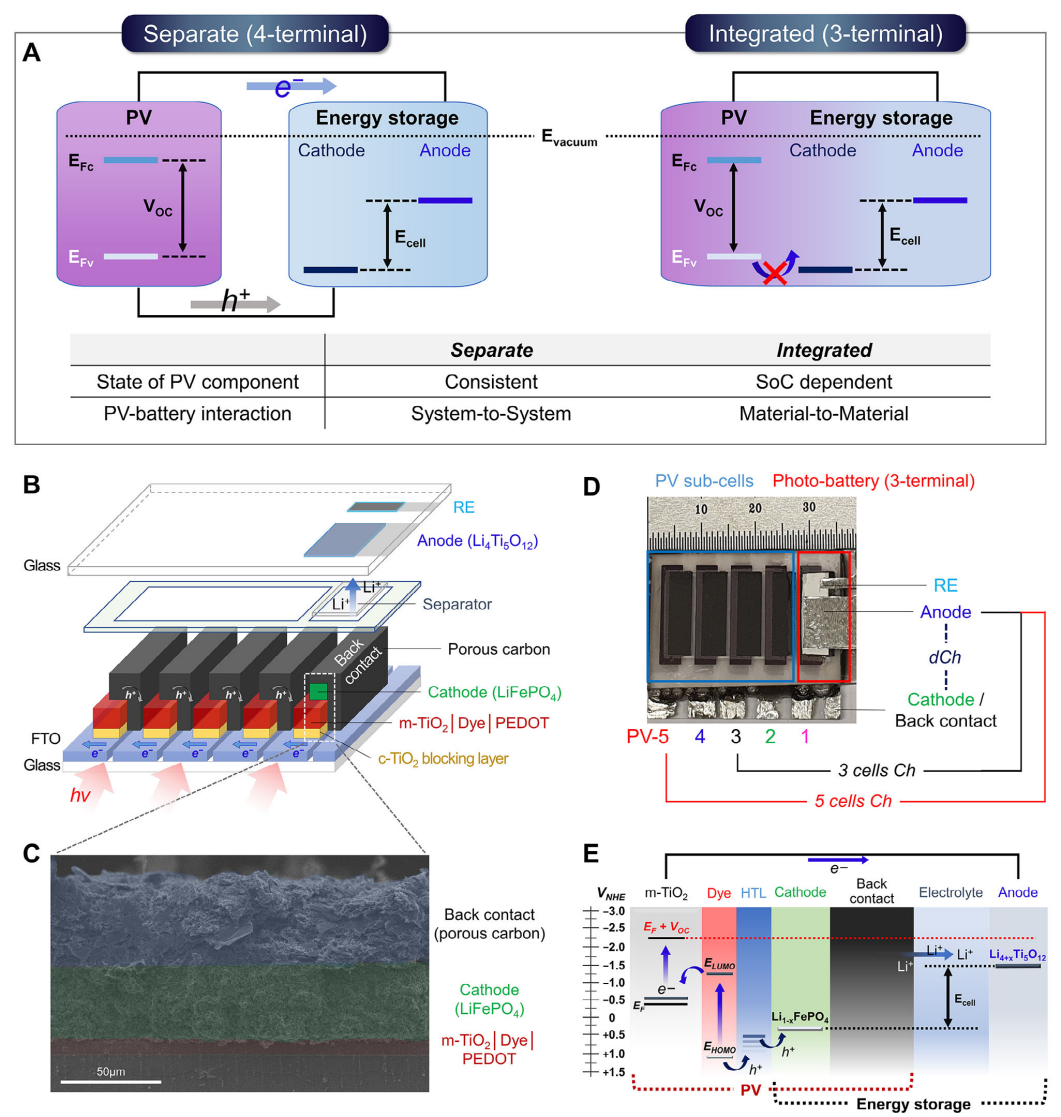
(A) Classification of photo‐rechargeable batteries: (left) separate 4‐terminal versus (right) integrated 3‐terminal designs, highlighting distinct internal energetics and PV‐battery interaction. (B) Schematic of the integrated PRB module. A reference electrode (same material as cathode) is incorporated for specific analysis. (C) Cross‐sectional SEM image of the cathode‐embedded PV cell. (D) Digital photograph of the assembled PRB module incorporating the 3‐terminal photo‐battery and serial PV sub‐cells; Note that different PV‐electrode is connected to an anode depending on the required photo‐charging voltage. For discharging, cathode and anode are connected with a constant current applied. (E) Energy diagram illustrating the operational mechanism, where serial PV sub‐cell coupling shifts the TiO_2_ quasi‐Fermi level (E_F_) by the cumulative photo‐voltage; E_F_ + V_OC_.

Demand for small off‐grid autonomous power supplies has rapidly increased along with the development of sensor networks for smart cities, intelligent farming, remote mining, and asset monitoring [1, 2]. Combined energy harvesting and storage systems are a promising technology for these applications [3, 4]. In particular, light is an attractive, ubiquitous energy source, but due to its intermittent nature, it needs to be combined with energy storage solutions such as rechargeable batteries. Highly integrated photo‐rechargeable batteries (PRB) are being pursued as compact stand‐alone solutions (Table S1) [5]. There have been many reports on photo‐charging systems, and they are classified into separate (four‐terminal) and integrated (three‐terminal or two‐terminal) designs (Figure 1A) [6, 7].

The separate four‐terminal designs consist of two independent systems [8, 9], which are representative of most solar energy installations, such as solar farms, because of their relatively low technical barrier. However, these may not be suitable for emerging autonomous devices, which favor compact designs. Further, solutions without power converters [10, 11] could lead to reductions in cost, volume, and energy losses between the harvesting and storage parts. For broad application, integrated PRBs need to be space‐efficient [12], operate under a range of different illuminances [13], and deliver a high output voltage [14]. Since a single unit photovoltaic (PV) cell provides a limited photo‐voltage (V_OC_) of around 1 V, serial PV module connections are needed to drive the charging process of most battery chemistries. For that reason, a few module types of integrated PRBs have been reported [15, 16, 17, 18], but to date, these do not meet all of the requirements listed above.

The defining distinction between the two designs lies in whether the PV and energy storage components are internally connected—a structural feature that profoundly impacts internal charge‐transfer physics. In the separate configuration, the PV and battery components are decoupled at the system level; thus, the PV operating state remains steady throughout the charging process, independent of the battery's state of charge (SoC). Conversely, the integrated architecture merges both components onto a single system, establishing direct material‐to‐material interfaces that inherently couple the PV state to the battery SoC. This intimate material‐to‐material coupling within the integrated design also governs the energetic alignment between the PV and battery components. Recently, our group demonstrated the physical condition required for photo‐induced electron transfer between the PV‐electrode and battery anode, showing that the quasi‐Fermi level of electrons must be positioned above the anode intercalation potential [19]. This principle also works for hole charge carriers. As depicted in Figure 1A, in separate configurations, photo‐charging should be possible, as long as the PV system generates higher V_OC_ than battery cell voltage (E_cell_). With the same materials in integrated configurations, however, photo‐charging is inefficient despite enough driving force for electron transfer if the hole quasi‐Fermi level is higher than delithiation potential of the cathode, which can vary with both the type of materials and the SoC of the battery.

In this work, we propose a compact PRB design consisting of integrated modules that can achieve photo‐charging, even under an indoor lighting condition. This design benefits from tunable charging voltages as well as consistent photo‐current thanks to horizontally arranged sub‐cells, rather than stacked type of PV modules [16, 18]. Using this system, we seek to better understand factors influencing the photo‐charging process as a function of SoC. Our results show two important phenomena: The PV current does not maintain its initial value as the SoC of battery increases due to worsening charge transfer at the hole transport layer (HTL)/cathode interface. Additionally, photo‐charging only proceeds when both charge carriers meet the energy level requirements for charge transfer to the battery reactions. These findings are different from classic separate PV–battery designs where both components do not interact and can help inform the rational design of integrated PRBs.

## 2 Results and Discussion

### 2.1 Device Configuration and Performance

Our PRB module configuration is shown in Figure 1B. It includes PV sub‐cells that are internally connected to a three‐terminal photo‐battery. Commercial organic dyes (WS72 and MS5, Dyenamo AB) [20, 21] were adopted as light harvesters given that they achieve high power conversion efficiencies under indoor light condition but also because they have lower HOMO level than the deintercalation potential of most cathode materials (Figure S1). The battery relies on a LiFePO_4_ (LFP) cathode and Li_4_Ti_5_O_12_ (LTO) anode, which is favourable for detailed charge kinetics studies thanks to their limited changes in potential profile with SoC (Figure S2). The devices were manufactured using a layer‐by‐layer process starting by spray coating a compact TiO_2_ film as a blocking layer [22]. Next, a mesoporous TiO_2_ film was screen‐printed as an electron transport layer as well as a scaffolding layer for dyes. A poly(3,4‐ethylenedioxythiophene) (PEDOT) HTL was grown in‐situ by photo‐polymerization [23]. All the cathode, anode, and porous carbon layers were deposited by linear knife‐over coating. A piece of anode (pre‐deposited on aluminum foil) was attached to a counter glass substrate. Both substrates were assembled in a sandwich configuration as shown in Figure 1B and tested in a 1 M LiTFSI in EC/EMC (3:7 vol%) electrolyte. All sub‐cells are internally connected in series, and the blocking layer in each cell prevents short‐circuit. As shown in the cross‐section SEM image of the PV (Figure 1C), the cathode layer is positioned directly on the HTL to improve the hole transfer to the cathode, which drives the cathode deintercalation (i.e. charging process). A porous carbon back‐contact on top of the cathode acts as the current collector for the battery operation as well as a counter‐electrode for the PV part. Given this layer's porosity, it does not prevent the Li‐ion transport needed for the battery operation. Since LTO has a more negative intercalation potential than the TiO_2_ quasi‐Fermi level (E_F_) [24], photo‐charging cannot occur with a single PV cell [19]. This energy level mismatch is solved by boosting our voltage using serial PV sub‐cells. The E_F_ of TiO_2_ can be boosted up by as much as the total V_OC_ of the PV sub‐cells, as described in the energy diagram (Figure 1E), overcoming limitations in previous designs in terms of delivered voltage, to make full photo‐charging possible without an external bias.

**FIGURE 2:**
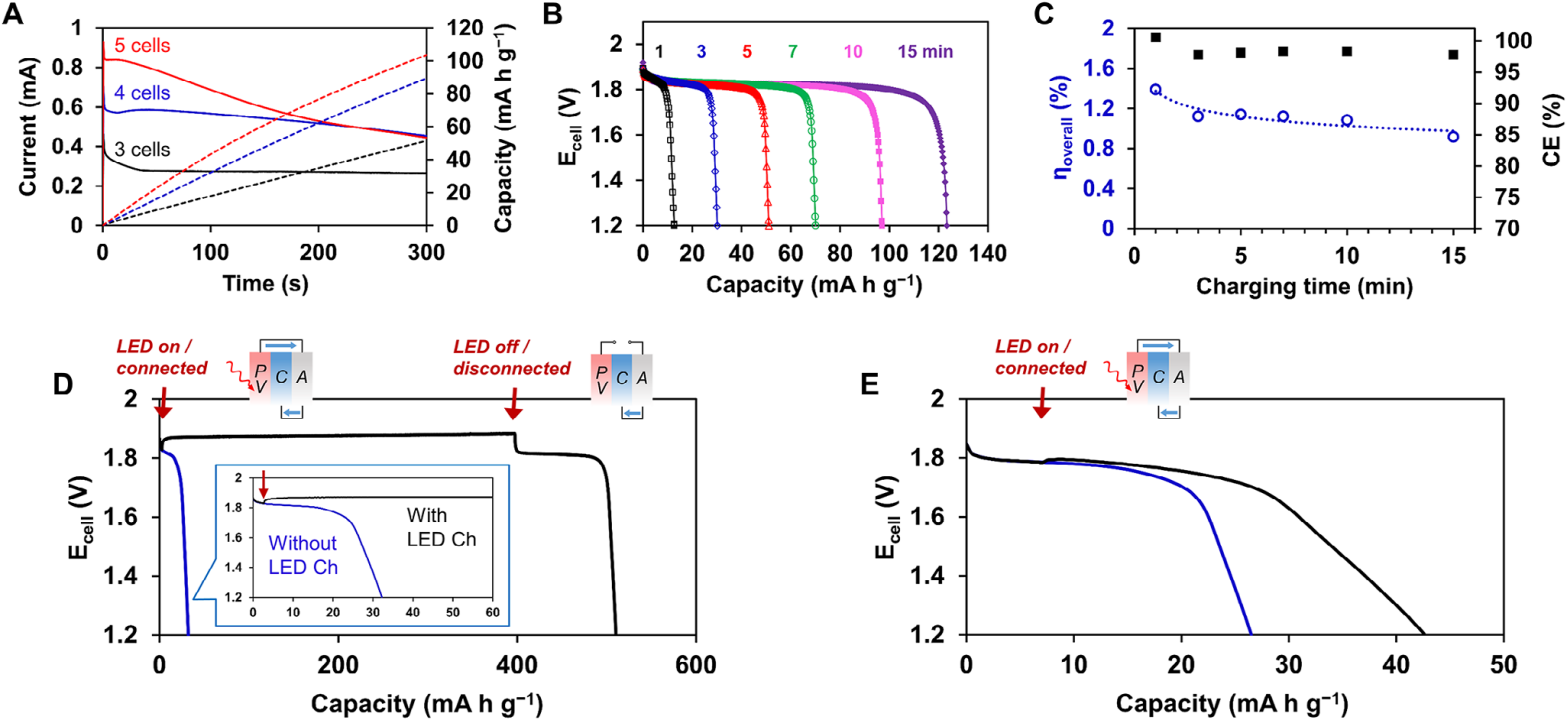
(A) Time‐dependent photo‐charging current (solid) and corresponding capacity (dashed) under 1‐sun illumination, varying the number of sub‐cell used. (B) Constant current discharge curves (51 mA g^−1^) following different 1‐sun photo‐charging duration and (C) corresponding η_overall_ and coulombic efficiencies. (D) Constant current discharge curves (32 mA g^−1^) following indoor LED lamp (1000 lux) photo‐charging, comparing standalone discharge (blue) against concurrent in situ photo‐charging (black). Note that discharge capacity is extended until the photo‐charging is stopped. (E) Discharge curves at accelerated rates (84 mA g^−1^) exceeding the photo‐charging rate.

The device performance was evaluated by photo‐charging under 1‐sun and subsequent constant current discharge. In these experiments, the last PV‐electrode where electrons are extracted, and the anode is connected for the photo‐charging (no bias voltage applied), while the battery anode and cathode are galvanostatically discharged to measure the stored capacity. Here, the PV‐electrode for the photo‐charging is decided according to the number of PV sub‐cells needed, as shown in Figure 1D. The number of PV sub‐cells used for the photo‐charging step allows to set the voltage to which the battery will be charged (upper cut‐off), and this needs to be optimized judiciously as it significantly affects the battery cycle life [25, 26]. The effect of charging voltage on battery capacity retention was tested using LFP**||**LTO full‐cell batteries without PV modules (Figure S3). To simulate the photo‐charging process, the cells were charged by applying constant voltages (2.5, 3.0, or 3.5 V) similar to the V_OC_ of PRB module with different numbers of sub‐cells used (Figure S4A), followed by a constant current discharge. We find that the coulombic efficiency (CE) is decreasing, and the shape of discharge curves is gradually distorted when the cell is cycled with a charging voltage of 3.5 V. In contrast, the cells retain almost 100% of their initial capacity with lower charging voltages. On the other hand, a higher voltage increases the driving force for photo‐induced electron transfer towards anode (or upper cut‐off voltage, see Figure S4B) and leads to higher photo‐charging current as shown in Figure 2A for integrated devices. To balance device lifetime while maintaining a decent photo‐charging current, we choose to use 3 PV sub‐cells and one photo‐battery unit for photo‐charging in the following experiments, unless stated otherwise.

The overall energy conversion and storage efficiency (η_overall_) is determined as η_*overall*_ =  (*E_out_*/*P_in_A_PV_t_ch_*) × 100, where E_out_, P_in_, A_PV_, t_ch_ are the output energy (J), incident light intensity (mW cm^−2^), total PV cell area (cm^2^), charging time (s), respectively [27]. Our best PRB device has a η_overall_ of 1.5% after 5 min of photo‐charging under 1‐sun illumination. After photo‐charging for different times, the PRBs are discharged, with a consistent CE around 98.5%, indicating that most of photo‐induced charges are stored electrochemically in the battery. Figure 2B shows the increase in capacity delivered by the battery as the charging time increases. We also note a decay in η_overall_ with longer charging times (Figure 2C), which is a result of decreasing charging currents with time as discussed further on. Our PRBs also operate under indoor light condition (white LED, 1000 lux, Figure S5). We investigate the effect of simultaneous light‐charging the PRB while being discharged. The PRB is photo‐charged using a desk LED lamp, and then its discharge curve is measured while simultaneously photo‐charging it under the same LED light. This is illustrated for the discharge current being either lower (32 mA g^−1^, Figure 2D) or higher (84 mA g^−1^, Figure 2E) than the photo‐charging current. In the case of low discharge currents (Figure 2D), the PV‐electrode delivers all the required current and the battery remains charged. In contrast, if the discharge current exceeds what is provided by the PV‐modules, the output voltage drops as the battery undergoes discharge, though the discharge rate is slower than in nonsimultaneous charging conditions (Figure 2E).

### 2.2 Photo‐Charging Current Decay According to Charge Mode

**FIGURE 3:**
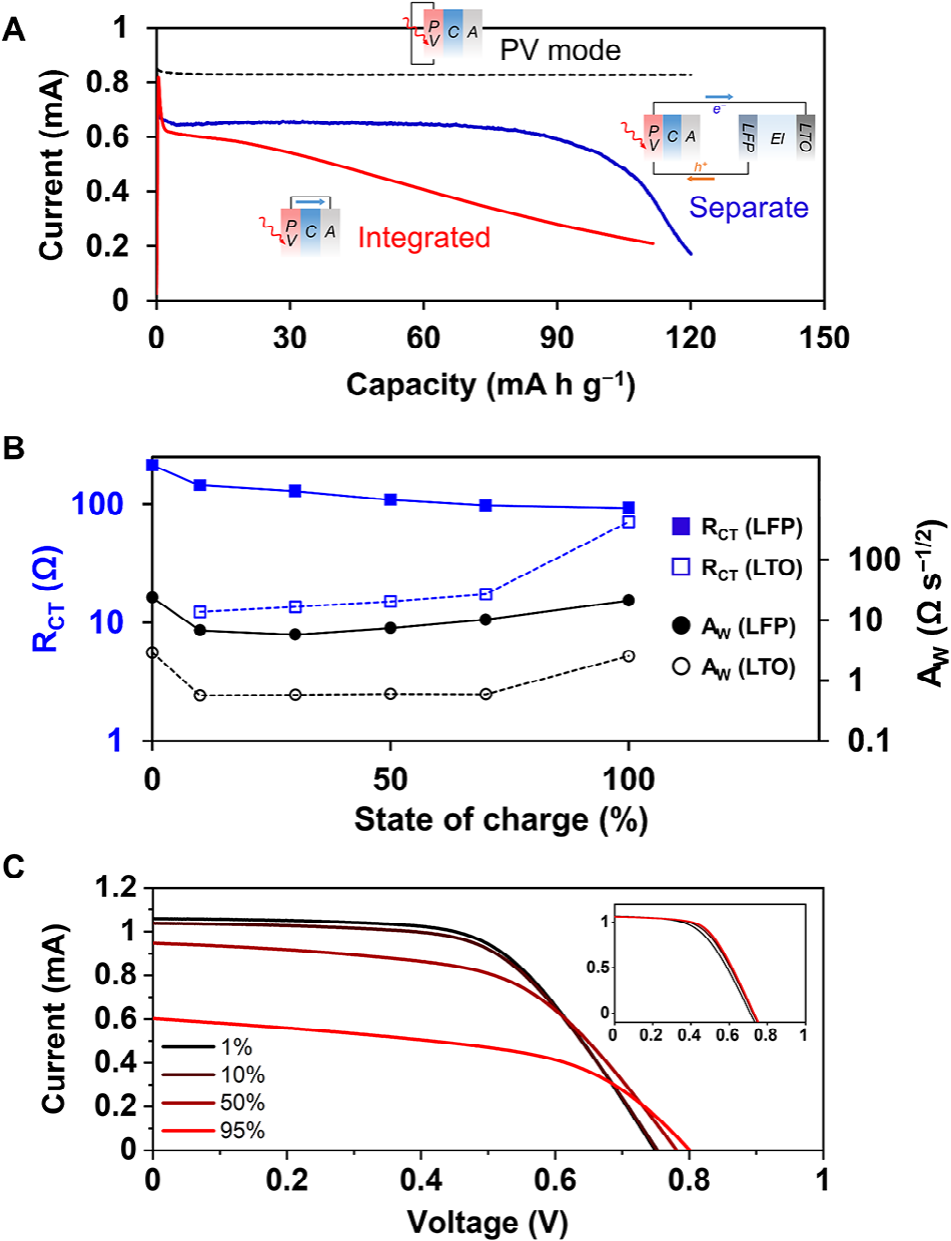
(A) Photo‐charging current curve of LFP**||**LTO battery depending on the charge mode. Both separate (blue) and integrated (red) architectures are shown as a function of photo‐charged capacity. Time dependent short‐circuit currents (PV mode) are plotted for comparison (black dashed). (B) SoC dependence of charge transfer resistance (R_CT_) and Warburg coefficient (A_W_) derived from LTO**||**Li and LFP**||**Li half‐cells. Increase of SoC denotes delithiation for LFP and lithiation for LTO, respectively. (C) SoC dependence of PV JV curves obtained from the photo‐battery unit. The inset shows the case of separate charge mode.

As shown in Figure 2A, the photo‐charging current decays over time, which may be the reason for the decrease in the η_overall_ with increasing photo‐charging time. Similar shapes of photo‐charging current curves were observed with different types and amounts of conducting carbon additive in the cathode layer (Figure S6). To further understand the origins of this behaviour, we compared the photo‐charging current curves (at 1‐sun) in integrated and separate modes (Figure 3A). Although the PV‐electrode can supply a constant current during the entire duration of illumination (dashed line in Figure 3A), the photo‐charging currents in both charge modes decrease over time. This decay is particularly pronounced in the integrated mode; in contrast, the charging current for the separate design remains consistent until roughly 80% SoC is achieved.

Due to the shared interfaces in the system, it is expected that integrated PRBs are influenced by more factors than separate designs. There is, however, a common factor. In both cases, the photo‐charging current can be influenced by battery impedance. To observe the SoC dependence of battery impedance, two distinct half‐cell configurations (LFP**||**Li and LTO**||**Li) were assembled using the respective cathode and anode materials. (Figure S7). The corresponding charge transfer resistance (R_CT_) and Warburg coefficient (A_W_) values are plotted as a function of SoC (Figure 3B). We find that the R_CT_ of LFP cell keeps reducing as the SoC of the battery increases, while other factors start increasing from 10% of SoC. These results agree with previous studies [28, 29]. Despite the decreasing R_CT_ of LFP, overall battery impedance is built up as the SoC increases, which is one of factors limiting the photo‐charging current. It is noticeable that resistances rise rapidly when SoC reaches about 80%. This explains the rapid decay of photo‐charging current in separate charge mode. However, it cannot fully explain the continuous current decay seen in integrated PRBs, suggesting that other factors are at play.

We also considered the thermodynamic driving force as an influential factor for photo‐charging current decay, which is defined as the potential gap between PV‐electrode and operating anode (Δ*E*_*PV* − Anode*_) during the photo‐charging process as shown in Figure S8A. We found that the Δ*E*_*PV* − Anode*_ remains relatively steady before the SoC of roughly 50%, whereas the photo‐charging current begins to decay from the initial SoC (see Figure S8 for details). This explicit mismatch confirms that the ∆E_PV‐Anode*_ is not the primary factor governing the current decay.

We next consider a changing PV performance as another factor influencing photo‐charging current decay in the integrated mode. We obtained PV JV curves from the photo‐battery cell as a function of SoC (Figure 3C). Significant changes are seen in photo‐current and fill factor. The short‐circuit current reduces by about 50% of initial value after full charge and the shape of the JV curve is distorted at the same time. This is in stark contrast to separate four‐terminal mode, where the changes in PV JV curves with SoC are negligible (inset of Figure 3C). The causes for these differences are investigated in the next section.

### 2.3 Limiting Factors for Photo‐Current Generation of PV‐Electrode

To investigate the changes in the PV JV curves in integrated PRBs, we consider how photo‐current is generated in dye solar cells. The external quantum efficiency (EQE(λ)) can be expressed by four processes [30]: The efficiencies of light harvesting (η_lh_(λ)), photo‐excited electron injection (η_inj_), photo‐oxidized dye regeneration (η_reg_), and charge carrier collection (η_cc_) in an order of sequence (Figure S9). The light harvesting efficiency (η_lh_) is mainly determined by the absorption coefficient of dye layer [31] while the injection efficiency (η_inj_) is affected by various factors, such as the driving force for electron transfer from the dye to the conduction band of TiO_2_ [32], surrounding polarity [33], and dye‐to‐dye interactions [34]. In our system, both η_lh_ and η_inj_ are unlikely to contribute to the SoC dependence of photo‐current, given that TiO_2_/dye interface is physically isolated from the cathode by a compact PEDOT.

Next, we study the charge carrier collection efficiency (η_cc_) through the transient photo‐current/photo‐voltage (TPC/TPV) method [35]. The charge carrier extraction time (τ_ext_), which is an indicative of η_cc_, can be estimated by τ_*ext*_ = 1/(1/τ_*trans*_ − 1/τ_*rec*_) , where τ_trans_ and τ_rec_ are time constants for photo‐injected electron transport and interfacial charge recombination, respectively. As shown in Figure S10, there is a small increase in τ_ext_ at 100% SoC, this change is not significant. This result implies that electron diffusion through the TiO_2_ network does not depend on the battery SoC, implying that the origin of photo‐current reduction may be from processes earlier than the charge carrier collection.

**FIGURE 4:**
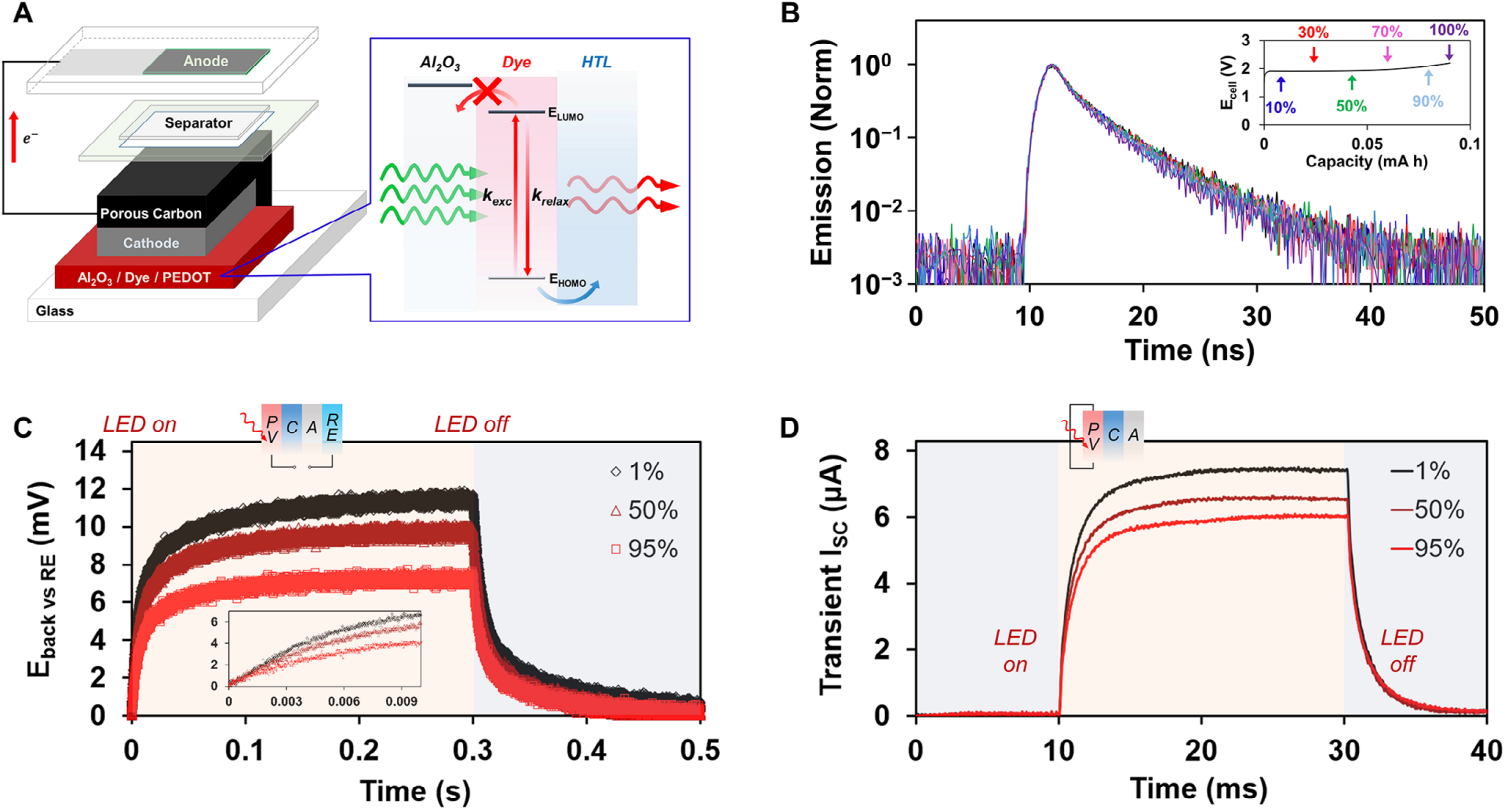
(A) Schematic of the operando TRPL study utilizing single photo‐battery architecture. Al_2_O_3_‐coated glass substrates were used instead of TiO_2_‐coated FTO substrate. (B) SoC‐dependent PL decay curves demonstrating negligible variation in photo‐induced hole transfer. (C) Transient photo‐voltage profiles of the back‐contact (E_back vs RE_) across varying battery SoCs. (D) Transient short‐circuit current (I_SC_) responses measured in PV mode at different battery SoCs.

Having ruled out several parameters, we suggest that inefficient dye regeneration could be responsible for the SoC dependence of photo‐current. The dye regeneration efficiency (η_reg_) is determined by the photo‐induced hole transfer at the dye/HTL interface, which can be evaluated by transient spectroscopic methods [36]. Following the photo‐induced hole transfer, photo‐injected hole transport through the HTL should be fast enough to maintain a consistent dye regeneration [37]. The interfacial hole transfer is studied by means of operando time‐resolved photoluminescence spectroscopy (TRPL) as illustrated in Figure 4A. A dummy cell was designed for operando TRPL analysis. Its configuration is the same as the photo‐battery cell, except that the TiO_2_ layer is replaced by Al_2_O_3_ to prevent photo‐excited electron injection (due to its wide bandgap nature). Consequently, a radiative exciton relaxation is primarily quenched by the photo‐induced hole transfer to the HTL (Figure S11). However, we do not find a noticeable change in PL decay as a function of SoC (Figure 4B). Considering that dye/PEDOT interfaces are mostly isolated from the cathode side, this seems to be a reasonable result. Instead, the PEDOT/cathode interface could be influenced by the battery operation, which we will discuss in the reflection analysis section.

We present a new approach to studying photo‐induced hole transport in our device. The TPV technique is employed again to track a potential change of back‐contact against a reference electrode. As illustrated in Figure S12A, when the device is illuminated, photo‐induced holes are injected from the dye to the HTL and move towards the back‐contact. This causes a small rise in back‐contact potential versus the reference‐electrode (E_back vs RE_). The number of photo‐induced charges at the back‐contact is proportional to ∆E_back vs RE_ as $Q\;\left(V\right)=\int_{0}^{V}C_{back}\left(V\right)dV\;$, where C_back_ is the differential capacitance at the HTL/cathode/back‐contact interface [38]. The validity of such methodology is confirmed by a control experiment (see Figure S12B,C for details). Now we observe the SoC dependence of the E_back vs RE_ (Figure 4C). Although we keep the intensity of pulse LED constant, E_back vs RE_ decreases with increasing SoC, and its trend agrees with the changes in PV JV curves described above. Also, the time constant of voltage rise (which is defined as the time taken for E_back vs RE_ to reach 63% of the final value) increases from 12.5 to 15.1 ms when fully charged. We attribute this to delayed photo‐injected hole transport. Despite consistent interfacial hole transfer at the dye/PEDOT (as indicated in the TRPL analysis), the hole transport efficiency does not keep pace with it at higher SoC. Consequently, the number of photo‐induced hole charge carriers reaching the back‐contact reduces as indicated by reduced E_back vs RE_. This result was double‐checked by impedance analysis (see Figure S13 for details).

We highlight that such change in photo‐induced charge kinetics is unlikely due to thermal effects. When a battery electrode is illuminated, its internal temperature can rise proportionally to the light intensity [39, 40, 41]. To minimize this light‐induced heat generation, the LED was turned on only for 20 ms with a low intensity (0.53 Mw cm^−2^), and the environmental temperature was controlled to 20°C. In these conditions, the temperature change in the device should be negligible, but we still measure a similar dependence of photo‐current on the battery SoC (Figure 4D). This result confirms that our observations are due to interactions between the PV and battery components rather than light‐induced heating effects.

### 2.4 Operando Spectroscopic Investigation of Interfacial Phenomena at HTL/Cathode

We believe that the retarded hole transport is mainly caused by phenomena at the HTL/cathode interface. In order to understand this, we carried out operando reflection spectroscopic study with an integrating sphere (Figure S14). Firstly, to understand the origin of spectral signals of cathode layer, we checked the reflectance of its individual components (LFP and conducting carbon mixture). As shown in Figure S14A, the spectrum of carbon‐only film is featured by a metallic infrared response. Its spectral shape is similar to that of cathode (LFP/carbon composite) film but shows higher plasmonic resonance characteristic (1000–1700 nm) [42, 43, 44, 45], which is attributed to higher proportion of the carbon. This suggests that the conducting carbon network is mainly responsible for the reflectance spectra of the cathode layer.

**FIGURE 5:**
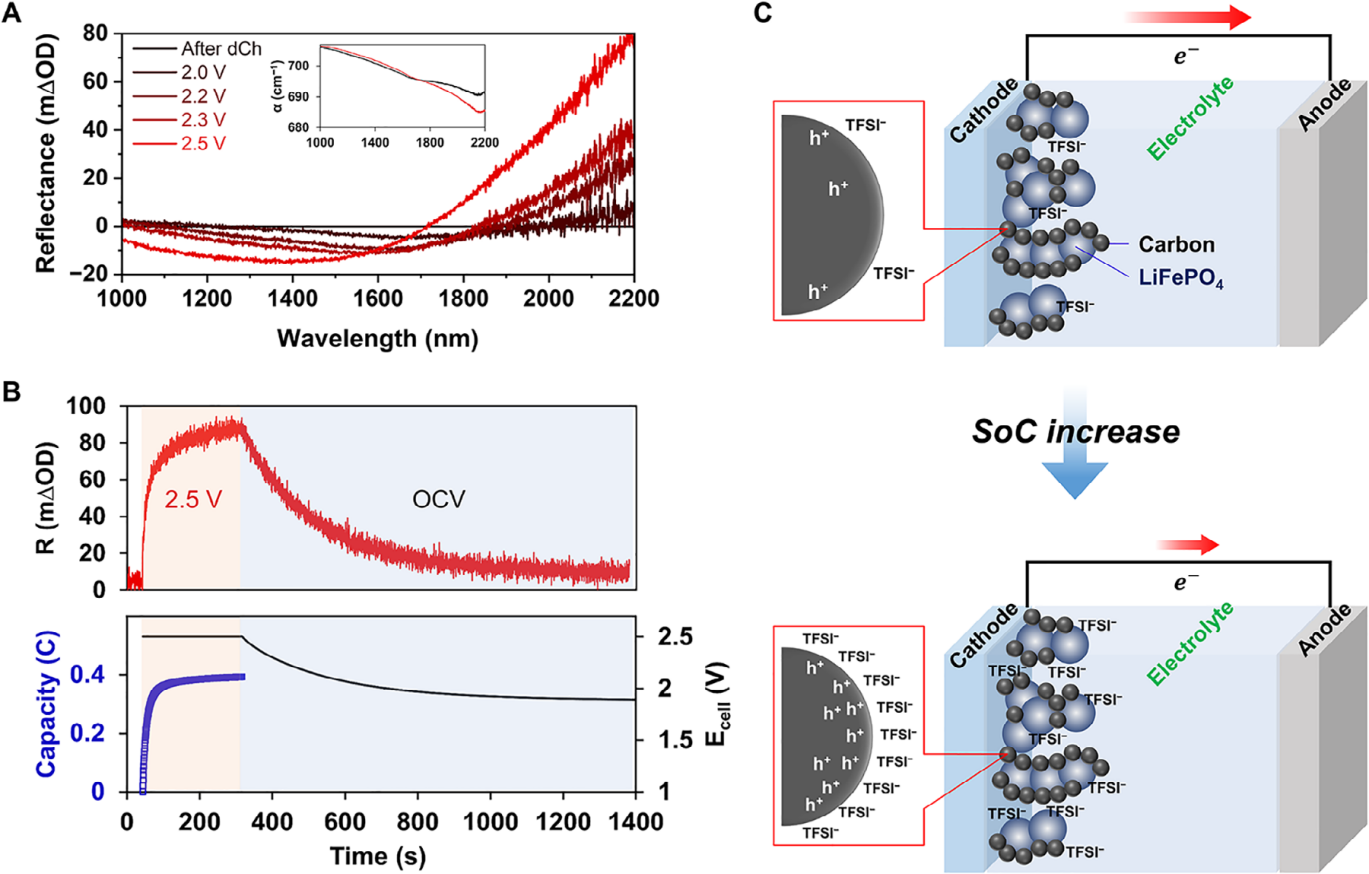
(A) Operando differential reflectance spectra of the LFP**||**LTO optical battery cell across varying SoCs (inset: wavelength‐dependent absorption coefficient, α). (B) Real‐time differential reflectance kinetics at 2150 nm (upper) and the corresponding capacity/cell voltage profiles (lower), showing synchronous optical and electrochemical trends. (C) Proposed mechanistic scenario from optical analysis: holes drive LFP delithiation under constant‐voltage charging, while advancing SoCs induce surface charge accumulation on the carbon network, triggering TFSI^−^ anion‐coupled capacitive charging.

An optical battery cell (LFP**||**LTO) was designed on a glass substrate to ensure light transmission (see Experimental section for details). Figure 5A shows differential reflectance spectra taken from the device (see Figure S14C for normal reflectance spectra). We find a competition between drop (shorter wavelength regime) and rise (longer wavelength regime) in reflectance when the charging voltage is applied. This is more obvious when a higher voltage is applied. In brief, both changes in reflectance suggest an increase of free charge carrier density on the surface of carbon network. To be more specific, the broad drop in reflectance (at 1000–1900 nm) could be attributed to an increased plasmonic oscillation of surface‐concentrated free charge carriers [46]. In contrast, the rise in reflectance (1700–2200 nm), which gets broader with higher voltage, can be explained by reduced interband optical transitions caused by a down‐shifted Fermi level [47, 48]. It is expected that the near‐infrared response of the SWCNT/graphene mixture in the cathode is mostly from the interband transitions over the spectral range of interest (0.56–1.24 eV) [49]. As for band‐to‐band transitions, the oscillation strength does depend strongly on the energetic position of free charge carriers [50, 51, 52]. Applying a charging voltage down‐shifts the electronic level of the cathode due to hole injection (as is the case in photo‐charging). Consequently, infrared light with lower frequency may not have enough energy to drive the interband transitions. This is clearly shown in a decrease of absorption coefficient (α) before and after bias (inset of Figure 5A), which is determined as α  =  2.303 × *A*/*d*, where *A*  =  (1 − *T* − *R)* is the absorbance, T is the transmittance (Figure S14B), R is the reflectance, and d is the cathode thickness [53].

We can better understand this mechanism through real‐time reflectance measurements (Figure 5B), where we monitor the peak wavelength of spectra (2150 nm) over time by applying a bias voltage. The reflectance rises abruptly when a 2.5 V charging voltage is applied, which is in good agreement with the time dependent capacity change. The reflectance drops again when the applied bias is removed. This reflectance decay curve is consistent with the open circuit voltage decay which is typical of ion release after capacitive charging [54]. Figure 5C illustrates the scenario proposed from our optical analysis. When a charging voltage is applied to the battery cell, the holes are injected into the cathode to drive delithiation. As the battery SoC increases, they accumulate on the conducting carbon network leading to an anion‐coupled capacitive charging. This phenomenon applies to the PEDOT/cathode interface in the photo‐battery cell. Given that the PEDOT network incorporates TFSI^−^ anions during the device fabrication (see Experimental section), the concentrated TFSI^−^ anions at the interface (which is induced by the hole accumulation at the cathode side) may disrupt the interfacial hole transfer. This is related to the changing PV JV curves along with battery SoC as evidenced by the transient photoelectrochemical analyses discussed previously.

### 2.5 Correlation of Energy Level Compatibility With Photo‐Induced Charge Transfer

As mentioned in Figure 1A, the energy level compatibility between electrode materials is important to design integrated PRBs. We have fabricated PRBs with different cathodes to investigate whether photo‐induced delithiation is influenced by the cathode potential (E_cathode_). The photo‐polymerized PEDOT HTL used in our system has a work function of around 5.0–5.2 eV [55]. This value is compatible with the delithiation potential of LFP as demonstrated above, but should be incompatible with certain cathode materials such as Li_1‐x_Mn_2_O_4_ (LMO). As shown in Figure S1, the delithiation potential of LMO is lower than the hole transport energy level through the PEDOT layer, which should make the hole transfer to the cathode delithiation inefficient.

**FIGURE 6:**
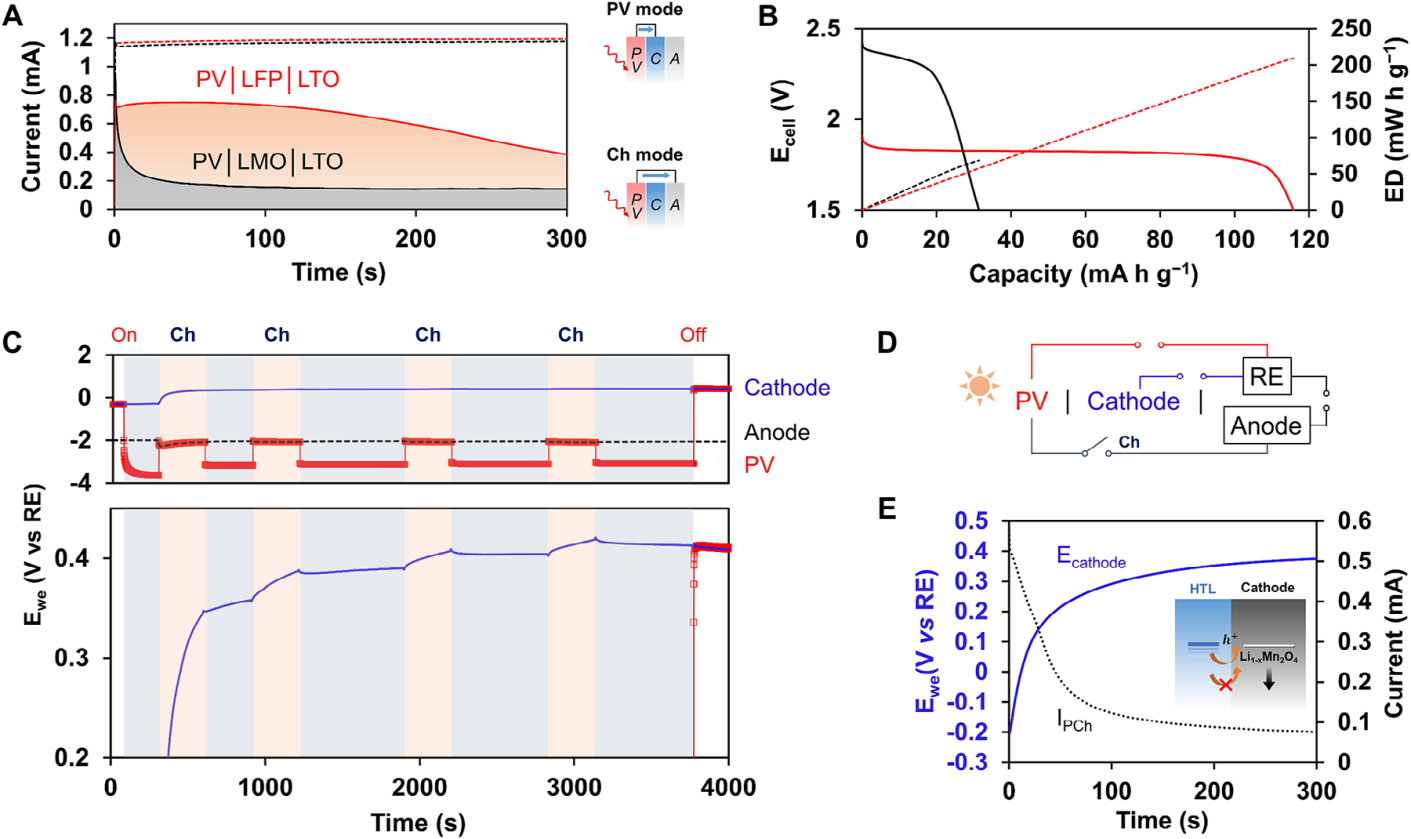
(A) Photo‐charging current curves (1‐sun) for different cathode materials, with time‐dependent PV currents (dashed) used to calculate photo‐charging current efficiency (η_PCh_). (B) Subsequent discharge curves (51 mA g^−1^) and corresponding energy densities (dashed), highlighting the superior energy density of the LFP PRB (209 mW h g^−1^ (90 mW h cm^−3^)) over the LMO PRB for an identical charging duration. (C) Potential tracking of three electrodes (PV‐electrode, cathode, and anode) against an LMO reference‐electrode in LMO**||**LTO PRB. Note initial change of cathode potential (blue). (D) Schematic of the electrode connections for the potential tracking experiment. PV‐electrode/anode connection is controlled by a switch for the alternate photo‐charging. (E) Cathode potential (E_cathode_) and corresponding photo‐charging current (I_PCh_) during the initial 5 min of photo‐charging (inset: proposed potential‐dependent interfacial charge kinetics for the LMO cathode).

The photo‐charging current under 1‐sun and subsequent discharge curves are compared with LFP and LMO cathodes (Figure 6A,B). Although both devices provide a stable short‐circuit current under PV mode, the photo‐charging current of the LMO PRB cell decays rapidly after the photo‐charging step begins as expected from the electrochemical potential diagrams (Figure S1). Even though a higher discharge voltage is achievable with LMO, the overall energy density is decreased due to lower attainable photo‐charge capacities. The corresponding photo‐charging current efficiencies (η_PCh_) are evaluated as η_*PCh*_ = (*Q_Ch_*/*Q_PV_* ) × 100, where Q_PV_ is the capacity obtained under PV mode and Q_Ch_ is the photo‐charged capacity. These values are determined for both cathodes using the area under the corresponding current curve [56]. The η_PCh_ of the LFP cell is 63.3%, whereas that of LMO cell is only 17.1% (Figure 6A), in agreement with the proposed inefficient photo‐induced delithiation of cathode.

To further understand the correlation between interfacial hole transfer and energy level matching, we monitored the potential change of three electrodes against a reference electrode during 5 min pulsed photo‐charging steps (Figure 6C,D). For this study, PV sub‐cells are fully used to ensure that sufficient driving force is theoretically present for electron transfer, as shown by the potential gap (about 1.1 V) between PV and anode. The PV potential sharply increases when the light is on. As the PV‐electrode and anode are short‐circuited during photo‐charging (orange shaded), the potentials of PV‐electrode and anode converge on the same value, which is indicative of electronic equilibrium [57]. We point out that the E_cathode_ shows a logarithmic growth. For the following photo‐charging steps, the rate of increment decreases. This initial outstanding change of E_cathode_ is in contrast with that observed in LFP**||**LTO PRBs (Figure S8C). As expected from the galvanic charge profile of LFP half cell (Figure S1), the LFP PRB cells do not show significant variations in E_cathode_, resulting in a negligible impact on the interfacial hole transfer. Figure 6E shows the opposite trend between E_cathode_ and photo‐charging current (I_PCh_) during the first 5 min of the photo‐charging step. These results suggest that the inefficient photo‐charging current of LMO PRB is related to the potential shift of LMO. As illustrated in inset of Figure 6E, the LMO potential is relatively negative right after discharge and shifts positively with increasing SoC, reducing the driving force for hole transfer. This mechanism is further corroborated by comparing the photo‐charging current profiles of LMO**||**LTO battery under different charge modes, following the experimental protocol from Figure 3A. As shown in Figure S15, the separate charge mode maintains a stable photo‐charging current until roughly 80% SoC, contrasting with the rapid current decay observed in the integrated configuration. This divergence provides compelling evidence of the internal energetic coupling inherent to true monolithic integration, which is absent in externally connected setups. These findings explain how the photo‐charging current rapidly decays despite sufficient photo‐charging voltage being available.

## 3 Conclusion

In summary, we have investigated the factors limiting the photo‐charging current in both separate and integrated designs of photo‐rechargeable batteries. We demonstrate that in the case of separate charge mode, changes in photo‐charging current are dominated by the battery impedance, which varies with SoC. In the case of integrated PRBs, our findings suggest that in addition to the battery impedance, other factors influencing the photo‐charging process include: (i) changes in photo‐current delivered by the PV‐electrode with battery SoC, which is attributed to the anion‐coupled charge accumulation between the HTL and conducting medium of the cathode, as verified by transient photoelectrochemical and operando spectroscopic analyses. (ii) Both charge carriers must meet the energy level requirements for driving cathodic and anodic battery reactions, with the delithiation potential of the cathode needing to remain above the quasi‐Fermi level of the HTL. These results show how to design new, more compact integrated PRBs that are promising for smart electronics.

## 4 Experimental Section

### 4.1 Cathode‐Embedded Solid State Photovoltaic Cell

The monolithic device was fabricated through a layer‐by‐layer process. Pre‐etched fluorine‐doped tin oxide (FTO glass, BIOTAIN CRYSTAL Co., Ltd.) was ultrasonically cleaned with acetone, ethanol, and deionized (DI) water for 30, 10, and 5 min, respectively. The cleaned FTO glass was pre‐heated on a hot plate at 505°C for 10 min. A compact TiO_2_ (c‐TiO_2_) layer was formed by spray pyrolysis at a flow rate of 50 µL s^−1^, keeping the distance between the airbrush gun nozzle (I.B.T, Republic of Korea) and the substrate at 30 cm. The precursor solution was prepared by diluting a titanium diisopropoxide bis(acetylacetonate) solution (Aldrich) with isopropanol in a ratio of 1:10 vol%. The sprayed FTO glass was annealed at 505°C for 60 min for crystallization. To minimize pinholes in the blocking layer, the FTO**|**c‐TiO_2_ substrate was dipped in a 40 mM aqueous TiCl_4_ solution (99.9% trace metals basis, Aldrich) at 75°C for 35 min. A mesoporous TiO_2_ (m‐TiO_2_) film was then deposited by screen‐printing a commercial paste to serve as an electron transport layer and a scaffold for the dyes. To achieve optimal impedance spectra, a mesoporous ZrO_2_ (m‐ZrO_2_) film was deposited by screen printing as an electrically insulating layer (helpful for reducing charge recombination). For the crystallization of the metal oxide films and the removal of organic additives, the screen‐printed substrate was annealed at 505°C for 60 min under air flow using a tube furnace (Carbolite Gero, Cadmus Products Ltd.). After cooling, the FTO**|**c‐TiO_2_ **|**m‐TiO_2_ **|**m‐ZrO_2_ substrate was immersed in the 40 mM aqueous TiCl_4_ solution at 75°C for 35 min once more, followed by a secondary annealing step at 500°C for 60 min.

The resulting FTO**|**c‐TiO_2_ **|**m‐TiO_2_ **|**m‐ZrO_2_ substrate was immersed in a dye solution at room temperature for 17 h; note that dye loading on the m‐ZrO_2_ layer is negligible compared to that on the m‐TiO_2_ layer. The co‐dye solution was prepared by blending WS72 and MS5 solutions in a 2:1 vol% ratio. The individual stock solutions were formulated as follows: 0.1 mM WS72 with 1.0 mM chenodeoxycholic acid in toluene/EtOH (1:4 vol%), and 0.1 mM MS5 with 1.0 mM chenodeoxycholic acid in t‐BuOH/CH_3_CN (1:1 vol%). PEDOT was utilized as the hole transport layer (HTL) and was synthesized in situ via photo‐polymerization [23]. For this step, a three‐electrode configuration was established using the FTO**|**c‐TiO_2_ **|**(m‐TiO_2_ **|**m‐ZrO_2_)@dye substrate as the working electrode, a Pt wire counter electrode, and an Ag/Ag^+^ reference electrode. The polymerization was performed in a CH_3_CN precursor solution containing 10 mM bis‐EDOT (ChemShuttle), 100 mM BMITFSI (Tokyo Chemical Industry UK Ltd.), and 100 mM LiTFSI (Fluorochem Ltd.). A constant voltage of 0.4 V (vs NHE) was applied to the working electrode—a potential at which polymerization does not spontaneously occur. Upon turning on the light‐emitting diode (LED), the reaction was initiated via photo‐induced hole transfer and allowed to proceed until the total extracted charge reached 20 µAh (35 mC cm^−2^). A post‐treatment step was implemented to enhance photovoltaic performance. A few drops of a CH_3_CN solution containing 20 mM LiTFSI and 200 mM 4‐tert‐butylpyridine (Aldrich) were cast onto the active area of the FTO**|**c‐TiO_2_ **|**(m‐TiO_2_ **|**m‐ZrO_2_)@dye@HTL substrate; after 1 min, the substrate was spin‐coated at 2000 rpm for 30 s and subsequently dried at 75°C for 15 min.

The cathode and porous carbon layers were sequentially deposited using a linear‐coating technique, followed by thermal drying at 80°C for 30 min in air. Aqueous cathode slurries were prepared by mixing the active material (LFP or LMO), carbon black as a conducting additive, and a carboxymethyl cellulose (CMC)/styrene‐butadiene rubber (SBR) binder mixture (1:1 wt%) in a weight ratio of 81:12:7. The resulting cathode layer exhibited an average thickness of 35 µm with a loading density ranging from 1.7 to 2.0 mg cm^−2^. The final configuration of the monolithic cell was defined as: FTO**|**c‐TiO_2_ **|**(m‐TiO_2_ **|**m‐ZrO_2_)@dye@HTL**|**cathode**|**back‐contact. The monolithic PV module consists of four PV sub‐cells coupled to a single photo‐battery unit, designated as [(PV sub‐cells)×4 **‒** Single photo battery] (Figure 1B). The architectural divergence between the PV sub‐cells and the photo‐battery is determined solely by the integration of the cathode layer:

1. PV sub‐cells: FTO|b‐TiO2|(m‐TiO2|s‐TiO2)@dye@PEDOT|back‐contact
2. Single photo battery: FTO|b‐TiO2|(m‐TiO2|s‐TiO2)@dye@PEDOT|cathode|back‐contact

### 4.2 LTO Anode and Photo‐Battery Assembly

Both MWCNT and GnP powders were separately dispersed in deionized (DI) water using CMC as a stabilizer through high‐energy sonication to achieve a gel‐like slurry. The two slurries were mixed to achieve a MWCNT@GnP slurry with a ratio of 1:1, followed by heating in a glycerol bath at 90°C while stirring to reduce the water content. Next, spherical LTO powder was mixed with the MWCNT@GnP slurry using an ARM‐310 Thinky mixer for 20 min. This LTO@MWCNT@GnP slurry was then coated onto a 17 µm thick aluminum foil and dried at 60°C on a hot plate for 1 h, followed by overnight drying in a vacuum oven at 60°C. The dried electrode had 40 µm of average thickness and 2.9 mg cm^−2^ of areal mass (mass ratio of 70 wt% LTO, 20 wt% MWCNT@GnP composite, and 10 wt% CMC).

A piece of anode and a piece of reference electrode were attached to a pre‐holed glass substrate to make the anode part. After overnight drying of the prepared substrate and a separator (hydrophilic PTFE membrane, pore size: 0.2 µm) in a vacuum oven (60°C), they were assembled in a sandwich configuration using thermoplastic films (Meltonix 1170‐60, Solaronix SA) at 110°C on a hot plate. The assembled photo‐battery cell was moved to a glove box to be filled with an electrolyte (1 M LiTFSI in EC/EMC (3:7 vol%)). The volume of electrolyte was estimated at around 23.8 µL. Finally, a hole for the electrolyte injection was dual‐sealed with Kapton tape and epoxy (RS Components Ltd.). As a secondary sealing, internal clearances of the photo‐battery cell were blocked by using epoxy to minimize leakage. The assembled PRB modules had an average total mass of 9.658 g.

### 4.3 Operando Time‐Resolved Photoluminescence Spectroscopy (TRPL)

TRPL spectra were recorded using a FLS1000 photoluminescence spectrometer (Edinburgh Instruments Ltd). A pulsed laser diode emitting at 512 nm served as the excitation source. Laser fluence was calibrated via an LED power meter, registering 30 mW cm^−2^ (3 nJ cm^−2^ per pulse) for 405 nm at a repetition rate of 10 MHz. The PL signals were captured with a peak wavelength of 645 nm.

To monitor the SoC‐dependent PL lifetime of the dye layer, a specialized dummy cell was fabricated. As illustrated in Figure 4A, its architecture mirrors the standard photo‐battery design, except that plain glass and an insulating mesoporous Al_2_O_3_ (m‐Al_2_O_3_) matrix were substituted for the FTO glass and electron‐transporting TiO_2_ layer, respectively. Because electronic charge extraction is prevented in this assembly, the PEDOT layer was in‐situ grown purely via photo‐induced hole transfer without potentiostat. Specifically, a glass**|**m‐Al_2_O_3_@dye substrate was illuminated under 0.2‐sun conditions for 2 h while immersed in the bis‐EDOT precursor solution. Robust connectivity between the resulting PEDOT network and the dye molecules was confirmed by the modulation of the baseline PL lifetime (Figure S11). Constant current (0.2C) was applied to drive the battery charge reaction, during which TRPL profiles were intermittently acquired at specific SoC intervals (0%, 10%, 30%, 50%, 70%, 90%, and 100%). We also tried to see if the PL lifetime was influenced when the battery was charged by a constant voltage (2.2 V), and could not see any change in the PL decay.

### 4.4 Operando Reflectance Spectroscopy (Integrating Sphere Mode)

An optical battery cell was fabricated specifically for the operando reflectance study. The cell was configured between dual glass substrates, utilizing a current‐collector‐free LFP cathode and an LTO anode on aluminum foil as follows: Glass**|**LFP@SWCNT@GnP**|**1 M LiTFSI in EC/EMC (3:7 vol%)**|**LTO‐Al**|**Glass (Figure S14C). To realize the current‐collector‐free cathode layer, a highly conductive SWCNT@GnP composite framework was adopted. The LFP@SWCNT@GnP slurry was prepared in the same manner as the LTO slurry, except that SWCNT and LFP were substituted for MWCNT and LTO, respectively. The resulting slurry was blade‐coated onto a glass substrate and dried at 80°C on a hot plate for 30 min. Concurrently, a piece of the LTO anode was secured to the opposing glass substrate using thermoplastic hot‐melt films. Both substrates were subsequently laminated together at 110°C on a hot plate. Following overnight drying in a vacuum oven, the assembled cell was filled with the organic electrolyte inside an inert glove box and hermetically completed via epoxy sealing.

Reflectance spectra were recorded on a Cary 7000 Universal Measurement Spectrophotometer (Agilent Technologies, Inc.) using the built‐in Diffuse Reflectance Accessory (integrating sphere). The 100% reflectance and 100% transmittance baseline were calibrated using a reference PTFE plate positioned at the reflectance port. Spectral data were acquired across a wavelength range of 1000–2200 nm in double beam mode with an integration time of 0.1 s and a slit band width of 2 nm. To simulate the photo‐charging profile during reflectance analysis, the optical cell was potentiostatically charged at constant voltages, with the reflectance spectra collected after maintaining each applied potential (1.8, 2.0, 2.2, 2.3, and 2.5 V) for 5 min.

## Author Contributions

B.‐M.K.: conceptualization. B.‐M.K. and M.D.V.: funding acquisition. B.‐M.K.: methodology. B.‐M.K., A.P., H.A., W.X., Y.H., and M.D.V.: investigation. A.P., H.A., S.D.S., and M.D.V.: resources. B.‐M.K.: writing – original draft. B.‐M.K., A.P., S.D.S., and M.D.V.: writing – review and editing. B.‐M.K. and M.D.V: supervision.

## Conflicts of Interest

The authors declare no conflicts of interest.

## Supporting information




**Supporting File**: adma74159‐sup‐0001‐SuppMat.docx.

## Data Availability

For the purpose of Open Access, the authors have applied a Creative Commons Attribution (CC BY) license to any Author Accepted Manuscript version arising from this submission. The data underlying this paper can be found at the University of Cambridge online repository [https://doi.org/10.17863/CAM.132263].
