# Supplementary material for: Operation Limits of Integrated Photo‐Rechargeable Batteries
Source: Adv Mater. 2026 Jul 17;38(48):e74159. doi: 10.1002/adma.74159 (PMC13508720; doi:10.1002/adma.74159)
Supplement: Supplementary file 1 [file ADMA-38-e74159-s001.docx]

**Electronic Supplementary Information (ESI)**

Operation limits of integrated photo-rechargeable batteries

Byung-Man Kim^1^, Arvind Pujari^1,2^, Hooman Abbasi^1^, Weidong Xu^3^, Yutong Han^3^, Samuel D. Stranks^2,3^, and Michael De Volder^1,*^

^1^Institute for Manufacturing, Department of Engineering, University of Cambridge, Cambridge CB3 0FE, UK

^2^Cavendish Laboratory, Department of Physics, University of Cambridge, Cambridge CB3 0HE, UK

^3^Department of Chemical Engineering and Biotechnology, University of Cambridge, Cambridge, UK

Corresponding Author

* E-mail: mfld2@cam.ac.uk (M.D.V)

**Experimental methods**

Materials

All materials were used as received with no purification unless otherwise noted. Organic dyes (WS72 (DN-F18) and MS5 (DN-F22)) are commercially available from Dyenamo AB (Sweden). Commercial metal oxide pastes, TiO_2_ (30NR-D, average particle size: 30 nm, Greatcell Solar Materials) and ZrO_2_ (Zr-Nanoxide ZR/SP, particle size: 20-40 nm mixed with 5 µm, Solaronix SA) were used to fabricate the active layer. Commercial Al_2_O_3_ paste (Al-Nanoxide A/SP, Solaronix SA) was used for the TRPL spectroscopic study. Commercial LiFePO_4_ (LFP, avg. 3-4 μm of particle size, MTIKOREA) and LiMn_2_O_4_ (LMO, avg. 6-7 μm of particle size, Aldrich) were used to fabricate the cathode layer. The LFP particles were coated with 2-5 nm carbon for higher conductivity. Commercial Li_4_Ti_5_O_12_ (LTO, BTR New Material Group Co., Ltd) was employed for the anode. The LTO powder is featured by clusters of primary particles (avg. 4-10 μm of cluster size). Commercial carbon paste (DM-CAP-4703S, Dycotec Materials Ltd) was employed for back-contacts. Single-walled carbon nanotubes (SWCNT, purity: > 96%, length: 20-55 µm, average diameter: 1.0 nm) powder was obtained from Nanografi Nano Technology (Türkiye). Multi-walled carbon nanotubes (MWCNT, purity: > 90%, average length: 1.5 µm, average diameter: 9.5 nm) powder was obtained from Nanocyl SA (Belgium). Graphene nanoplatelets (GnP, 2-10 nm thickness, average diameter: 5 µm) powder was obtained from Thermo Fisher Scientific.

Photo-charging/discharging protocol

The photo-charging current was measured in chronoamperometry mode with 0 V applied between the PV electrode and the anode, corresponding to the short-circuit state under illumination. Subsequently, a constant current was applied between the anode and cathode for discharge while the battery cell voltage was recorded. All electrochemical measurements were carried out on a Biologic VMP3 potentiostat (Bio-Logic Science Instruments Ltd). Air mass 1.5 conditions (1-sun, 100 mW cm^−2^) were generated using a solar simulator (Newport Oriel LSH-7320). Indoor light evaluation was conducted using a white LED desk lamp (Philips N.V.) inside a custom-built black box, where illuminance was verified using a spectrometer (OHSP-350, Hangzhou Hopoo Light & Color Technology Co., Ltd.). The photo-active area was controlled by a 3D-printed black mask (18 mm^2^ and 37 mm^2^ for the 1-sun and indoor tests, respectively).

Electrochemical characterization

All electrochemical measurements were carried out on a Biologic VMP3 potentiostat. The photo-battery modules were tested at 20 °C with 45% relative humidity, while the coin battery cells were operated in an environmental chamber (25 °C / 34% relative humidity). Linear sweep voltammetry was performed under 1-sun illumination at a scan rate of 50 mV s⁻¹ to obtain the PV J–V curves of the photo-battery module. For the battery cycle tests, the charge/discharge process was carried out using two protocols: constant-current charge/constant-current discharge and constant-voltage charge/constant-current discharge, with the latter used to reproduce the photo-charging/discharging process. The reported average discharge capacities and their corresponding average Coulombic efficiencies were determined using data collected from three independent coin cells and three distinct PRB devices, respectively. The C-rate was calculated based on the theoretical capacity of the cathode material (170 mAh g⁻¹ for LFP). Electrochemical impedance spectra were recorded by applying a 10 mV sinusoidal pulse superimposed on a DC bias equivalent to the open-circuit voltage (OCV) over a frequency range from 10^6^ to 10^−1^ Hz. All impedance results were fitted with suitable equivalent circuits using the ZView software (Scribner Associates, Inc.). To determine the highest occupied molecular orbital (HOMO) level of the organic dyes, cyclic voltammetry was carried out at scan rates of 10–50 mV s⁻¹ in a three-electrode setup including the FTO**|**TiO_2_@dye substrate as the working electrode, a Pt wire as the counter electrode, and Ag/Ag^+^ as the reference electrode in a supporting electrolyte (100 mM LiTFSI in CH_3_CN). All potentials were converted to the normal hydrogen electrode (NHE) scale by internally adjusting the zero point with ferrocene/ferrocenium (+0.63 V vs. NHE).

Transient photo-current/photo-voltage (TPC/TPV)

Chronoamperometry and OCV measurements (for TPC and TPV, respectively) were performed on a VMP3 potentiostat equipped with EC-Lab Express software for fast data acquisition. The minimum data intervals are 20 μs for the OCV mode and 24 μs for the chronoamperometry mode, which are sufficient to study the charge collection dynamics in the dye-sensitized solar cells. Two white LEDs (MCWHL8, pulse rise/fall time: ~5 μs, Thorlabs, Inc.) were utilized as background and pulse light sources, which were controlled by the potentiostat. To avoid underestimating the electron diffusion/charge recombination times, the pulse intensity was controlled to keep the TPC peak value below 10% of the background current and the TPV peak voltage below 10 mV. As for the photo-induced hole transport analysis (**Figure S12**), a longer pulse duration (300 ms) was selected to maximize the ∆E_back vs RE_ value and allow the potential rise curves to stabilize.

**Table S1.** Comprehensive performance benchmarking of state-of-the-art integrated photo-rechargeable batteries. This compilation strictly includes fully light-rechargeable architectures with verified, integrated operating mechanisms, excluding external connection of discrete photovoltaic–battery systems.

| Number of electrode | Active materials & structure | Electrolyte | Photocharging condition | Output voltage range (V) | CE^[a]^  (%) | η_overall_^[b]^  (%) | Refs. |
| --- | --- | --- | --- | --- | --- | --- | --- |
| 3 | TiO_2_-WS72-PEDOT **\|** LiFePO_4_ **\|\|** Li_4_Ti_5_O_12_ | 1.0 M LiTFSI in EC/EMC (5:5 vol%) | Solar simulator  (100 mW cm^−2^) /  Desk LED lamp  (0.35 mW cm^−2^) | 1.2 ~ 2.4 | 96 ~ 99 | 1.5 (100 mW cm^−2^) /  2.6 (0.35 mW cm^−2^) | This work |
| 2 | CdS@Pt **\|** Li_2_S_4_+LiOH(aq) **\|** membrane **\|\|** Li metal | 1.0 M LiClO_4_ in EC/DMC (5:5 vol%) | Natural sunlight (11:00 a.m. to 1:00 p.m.) | 2.3 ~ 2.6 | - | Li metal keeps being consumed over cycles | ^[9]^ |
| 3 | TiO_2_-N719 **\|** Iˉ/I_3_ˉ **\|** membrane **\|** S_4_^2−^/S_2_^2−^ | Catholyte: 1 M I_2_ + 0.1 M NaI + 1 M NaClO_4_ /  Anolyte: 1 M Na_2_S + 3 M S + 1 M NaClO_4_  in MeCN/THF (2:1 vol%) | Solar simulator  (100 mW cm^−2^) | 0.2 ~ 0.8 | ~ 100 | - | ^[10]^ |
| 2 | MoO_3_ **\|\|** MnO_2_ | 1.0 M Na_2_SO_4_ (aq) | UV illumination by a 300 W Xe lamp  (200 mW cm^−2^) | 0.2 ~ 0.6 | - | - | ^[11]^ |
| 2 | LiFePO_4_-N719 **\|\|** Li metal | 1.0 M LiPF_6_ in EC/DEC (3:7 vol%) + 2wt% VC | Solar simulator  (100 mW cm^−2^) | 2.8 ~ 3.4 | - | 0.06 ~ 0.08 | ^[12]^ |
| 2 | 2D cyanamide-polyheptazine  imide **\|\|** glassy carbon (ref. Ag/AgCl) | 1 M Na-based phosphate buffer solution (pH 7) + 4-methylbenzyl alcohol | Solar simulator  (100 mW cm^−2^) | −0.8 ~ −0.2 *vs* Ag/AgCl | 75 ~ 80 | - | ^[13]^ |
| 3 | TiO_2_-Z907 **\|** Iˉ/I_3_ˉ **\|** membrane **\|\|** Fe_2_(MoO_4_)_3_ | 1.0 M NaClO_4_ in PC | Solar simulator with a 500-1100 nm filter (100 mW cm^−2^) | 0 ~ 0.4 | - | - | ^[14]^ |
| 3 | Rutile TiO_2_ NW **\|** Iˉ/I_3_ˉ **\|** membrane **\|** Br_2_/Brˉ | Catholyte: 1.2 M KBr + 0.5 M LiH_2_PO_4_ (aq) /  Anolyte: 1 M KI + 0.1 M I_2_ + 0.5 M LiH_2_PO_4_ (aq) | Solar simulator  (100 mW cm^−2^) | 0 ~ 0.5 | 97 ~ 100 | 0.16 | ^[15]^ |
| 3 | TiO_2_-N719 **\|** Iˉ/I_3_ˉ@PEO **\|** membrane **\|\|** 9,10-anthraquinone | LiClO_4_ + EC + PC + PEO (Mw=100000) | Solar simulator  (100 mW cm^−2^) | 0 ~ 0.75 | - | - | ^[16]^ |
| 3 | TiO_2_-MAPbI_3_-Carbon **\|** Sulfur@CNT **\|\|** Li metal | 1.0 M LiTFSI + 0.1 M LiNO_3_ in DOL/DME (5:5 vol%) | Solar simulator  (100 mW cm^−2^) | 1.7 ~ 2.3 | - | 5.14 | ^[17]^ |
| 3 | TiO_2_-Y123 **\|** Cu(dmp)_2_ **\|** membrane **\|\|** LiMn_2_O_4_ | 0.8 M LiClO_4_ in CH_3_CN | Solar simulator  (100 mW cm^−2^) /  Desk LED lamp  (0.06 ~ 0.6 mW cm^−2^) | 0 ~ 0.8 | ~ 100 | 0.53 (100 mW cm^−2^) /  11.5 (0.15 mW cm^−2^) | ^[18]^ |
| 3 | (FAPbI_3_)_0.83_(MAPbBr_3_)_0.17_@Si tandem **\|** BTMAP-Vi **\|** membrane **\|** N^Me^-TEMPO | 0.1 M N^Me^-TEMPO as anolyte and 0.1 M BTMAP-Vi as catholyte in 1.0 M NaCl as supporting electrolyte | Solar simulator  (100 mW cm^−2^) | 0.8 ~ 1.5 | ~ 99 | 20.1^[c]^ | ^[19]^ |
| 3 | TiO_2_-Cs_0.15_FA_0.85_PbI_3_ **\|** Joint carbon paper@P-NiCo_2_O_4_ **\|\|** Zn metal | 3 M KOH + 0.05 M Zn(Ac)_2_ (aq) | Solar simulator  (68.5 mW cm^−2^) | 1.4 ~ 1.9 | - | 6.4 | ^[20]^ |
| 3 | TiO_2_-dye **\|** Brˉ/Br_3_ˉ **\|** membrane **\|** *h*-WO_3_ | 0.1 M LiBr + 0.6 M DMPIBr +  0.025 M Br_2_ + 0.3 M 4-tert-butylpyridine | Solar simulator  (100 mW cm^−2^) | 0 ~ 0.9 | - | - | ^[21]^ |
| 2 | Ag **\|** ox-COF **\|\|** re-COF **\|** TiO_2_ | 1.0 M H_2_SO_4_ | Solar simulator  (100 mW cm^−2^) | 0 ~ 0.7 | - | 6.9 | ^[22]^ |
| 2 | PbPA-CsPbBr_3_ **\|\|** Li metal  (Cathode: $2{Li}^{+}+2e^{-}+O_{2}\to{Li}_{2}O_{2}$) | PVDF-based polymer (LiTFSI) | Solar simulator  (100 mW cm^−2^) | 1.0 ~ 3.0 | - | 0.72 | ^[23]^ |
| 2 | Cu_2_O-TiO_2_ **\|\|** Li metal  (Photoelectron from Cu_2_O induces lithiation of TiO_2_) | 1.0 M LiPF_6_ in EC/DEC (5:5 vol%) | White LED (no UV irradiation)  (100 mW cm^−2^) | 1.6 ~ 2.5 | - | 0.29 | ^[24]^ |
| 2 | ZnIn_2_S_4_ **\|\|** membrane **\|\|** Zn metal **\|\|** ZnIn_2_S_4_ | 1 M KOH (pH = 13.6) for photoelectrode  2 M ZnSO_4_ (pH = 4.0) for Zn anode | Solar simulator  (100 mW cm^−2^) | 0 ~ 1.26 | - | 1.45 | ^[25]^ |
| 2 | VS_4_-Biomass Carbon **\|\|** Zn metal | PVDF-HFP@Zn(CF_3_SO_3_)_2_ gel | Solar simulator  (100 mW cm^−2^) | 0.25 ~ 0.92 | - | 0.52 | ^[26]^ |
| 2 | NbWO_6_ **\|\|** Au (ref. Ag/AgCl) | 1 M LiCl + MeOH (10 vol%) (aq) | 365 nm UV LED  (206 mW cm^−2^) | −0.8 ~ −0.1 *vs* Ag/AgCl | - | 0.48 | ^[27]^ |
| 2 | SnO_2_-MoS_2_ **\|\|** Zn metal  (O_2_ reduction on cathode) | 2 M ZnSO_4_ (aq, pH 4) | 365 nm UV LED  (30 mW cm^−2^) | 0.2 ~ 1.0 | - | 1.0 | ^[28]^ |
| 2 | Fiber cell (Fullerene plasma-induced carbon clusters@polyaniline **\|\|** Zn metal) | ZnCl_2_ + NH_4_Cl in PVA-based gel (aq, pH 4) | Solar simulator  (100 mW cm^−2^) | 0.7 ~ 1.2 | - | 1.15 | ^[29]^ |
| 2 | BiOI@MWCNT **\|\|** Zn metal | 2 M Zn(OTf)_2_ (aq) | 470 nm LED  (11 mW cm^−2^) | 0 ~ 0.74 | - | 1.3 | ^[30]^ |
| 2 | MoSe_2_ **\|\|** Na metal  (Photoelectron induces CEI formation) | 1.0 M NaPF_6_ in EC/DMC (5:5 vol%) | 4 W indoor LED bulb  (11.3 mW cm^−2^) | 0.5 ~ 1.7 | - | 0.19 | ^[31]^ |
| 2 | 2D naphthalenediimide COF **\|\|** Pt wire (RE: Ag/AgCl) | 10mM 4–MBA (aq) (or with 1M MCl; (M = Li, Na, K, Rb, Cs)) | Solar simulator  (100 mW cm^−2^) | −0.62 ~ 0  *vs* Ag/AgCl | - | 0.012 | ^[32]^ |
| 2 | Ti **\|** BQ-TF COF@Ag **\|\|** TA-PT COF@CdS **\|** Ti | 1M H_2_SO_4_ (aq) | Solar simulator  (100 mW cm^−2^) or  380 nm LED  (0.2 mW cm^−2^) | 0 ~ 1.0 (single)  1 ~ 5  (5 tandem) | - | 12.1^[d]^ | ^[33]^ |

^[a]^ Coulombic efficiency (CE) of photo-rechargeable batteries is defined as $CE=({Q_{dCh}}/{Q_{PCh}})\times100$, where Q_dCh_ is the discharged capacity and Q_PCh_ is the photo-charged capacity.

^[b]^ Overall energy conversion/storage efficiency is defined as $\eta_{overall}=\frac{E_{out}}{P_{in}\times A_{PV}\times t_{ch}}\times100$, where E_out_, P_in_, A_PV_, t_ch_ are the output energy, incident light intensity, total PV cell area, charging time, respectively.

^[c]^ This work reports the solar-to-output electricity efficiency (SOEE) as below^[19]^:

$SOEE=\frac{I_{PV}\times V_{RFB}\times CE\times VE}{P_{in}\times A_{PV}}\times100$, where I_PV_ is the operating photocurrent provided by the PV-electrode, and V_RFB_, CE and VE are the cell voltage, coulombic efficiency and voltage efficiency of the flow battery, respectively.

^[d]^ This work reports the solar-to-electrochemical energy storage efficiency (η_SES_) as below^[33]^:

$\eta_{SES}=\frac{\Delta P_{light/dark}}{P_{light}}=I\times\frac{\Delta V}{P_{light}}$, where ∆P_light/dark_ is the difference in output power between illuminated and dark conditions, and P_light_ is the output power under illumination, respectively.

**Figure S1.** Cyclic voltammogram (left y-axis) of organic dyes adopted in our system (WS72 and MS5) and galvanostatic charge/discharge curves (right y-axis) of cathode materials (LFP and LMO). Ferrocene was used as an internal reference (+0.63 V vs NHE) for CV measurement. Potential of cathode materials is presented versus normal hydrogen electrode by considering the reduction potential of lithium (-3.04 V vs NHE). Range of PEDOT work function is also presented (sky-blue shaded) according to reference.^[6]^

**Figure S2.** (A) Galvanostatic charge/discharge profiles (0.1C) of battery half- and full-cells with active materials adopted in PRB module (LFP and LTO). (B) Galvanostatic charge/discharge profiles (0.1C) of LFP**||**LTO full-cells obtained from PRB module (red plots) and coin cell (black plots). Initial cycles are presented alongside. (C) Average discharge capacities (bars, left axis) and corresponding Coulombic efficiencies (plots, right axis) evaluated across three independent coin cells (left) and three distinct PRB modules (middle and right), respectively. The red bar exhibits the average discharge capacity delivered after photo-charging (1-sun).

**Figure S3.** (A) Discharge capacity retention of LFP**||**LTO full-cell. Experimental protocol is as follows: constant voltage charge (2.5 V or 3.0 V or 3.5 V)/constant current discharge (0.3C). Corresponding discharge curves (1st, 40th, 80th) are shown according to applied charge voltage: (B) 2.5 V, (C) 3.0 V, and (D) 3.5 V. Note that the shape of discharge curve is deformed with high charge voltage (3.5 V).

**Figure S4.** (A) (Left) PV JV curves of PRB module with different number of sub-cells connected. The photo-voltage is proportional to the number of sub-cells and photo-current remained similar by serial effect. (Right) According to the number of sub-cells needed for measurement, corresponding PV-electrode is connected to back contact (or counter electrode). For example, 4th PV-electrode is used for 4 cells measurement. (B) Energy diagram describes that driving force for electron transfer (or upper cut-off voltage) is proportional to the number of sub-cells used for photo-charging.

**Figure S5.** Spectral power density of LED lamp (Philips N.V.) used for an indoor performance evaluation: 500 lux (0.181 mW cm^−2^) and 1000 lux (0.356 mW cm^−2^).

**Figure S6.** Photo-charging current density (J_PCh_) curves obtained from the LFP||LTO PRB modules with different cathode conditions. Note that similar decay shapes of J_PCh_ are shown with different ratio of conducting carbon additive (Super P) as well as different type of conducting additive (single-wall carbon nanotube@graphene nanoplatelet composite).

**Figure S7.** SoC dependent impedance spectra obtained from battery half-cells: LFP**||**Li (left) and LTO**||**Li (right). Bode plots (lower) show the frequency regime for sequential charge kinetics^[1-2]^: current collector/cathode contact resistance (ω_cont_), SEI-diffusion process of lithium anode (ω_SEI_), charge transfer at the cathode/electrolyte interface (ω_CT_), and Warburg impedance (ω_W_). Increasing SoC denotes delithiation for LFP and lithiation for LTO, respectively. Charge transfer resistance (R_CT_) and Warburg coefficient (A_W_)^[7-8]^ were extracted from the corresponding frequency regimes, ω_CT_ and ω_W_, respectively.

**Figure S8.** (A) Real-time potential tracking of three electrodes (PV-electrode (red), cathode (blue), and anode (black)) versus an LFP reference-electrode in LFP||LTO PRB. The potential gap (∆E_PV-Anode*_ = E_PV_ − E_Anode*_) between PV-electrode (E_PV_) and operating anode (E_Anode*_) defines the thermodynamic driving force for light-induced electron transfer. (B) Corresponding photo-charging current density (J_PCh_) profile of the integrated LFP||LTO PRB system, exhibiting early-stage current decay despite a sustained thermodynamic driving force. (C) Identical real-time potential tracking with the magnified cathode potential profile (blue line). The cathode potential slightly increases during photo-charging. The maximum change of cathode potential is about 45 mV over 90% SoC. (D) Schematic of potential tracking experiment. OCV of three electrodes are monitored against the RE while PV-electrode/anode connection is controlled using a switch.

We monitored the real-time evolution of the thermodynamic driving force between the PV-electrode and the anode during the photo-charging (**Figure S8A**). For that, open circuit voltages of three electrodes were separately tracked against the LFP reference electrode (RE). Upon illumination, the PV potential (E_PV_) shifts abruptly toward negative values. When the PV and anode components are short-circuited for the photo-charging (orange shaded), their potentials closely converge, establishing electronic equilibrium. In this state, the potential gap (indicated by the red double-headed arrows) between PV-electrode (E_PV_) and operating anode (E_Anode*_) defines the thermodynamic driving force (∆E_PV-Anode*_) for light-induced electron transfer. The initial potential gap stands at approximately 400 mV and begins to narrow from the SoC of roughly 50%, eventually reaching 130 mV at full charge.

However, correlating this potential profile with the corresponding photo-charging current density (J_PCh_) over time (**Figure S8B**) reveals a prominent discrepancy: the photo-charging current begins to decay from the initial SoC, even while the thermodynamic driving force remains high. This explicit mismatch confirms that the macroscopic potential gap is not the primary limiting factor for the current decay.

**Figure S10.** (A) Transient photo-current and (B) photo-voltage decay curves where electron transport (τ_trans_) and charge recombination times (τ_rec_) are determined. Decay half times^[5]^ were used to determine time constants. (C) Charge carrier extraction time (τ_ext_) is estimated by $\tau_{ext}=1/{(1/{\tau_{trans}}-1/{\tau_{rec}})}$.

**Figure S9**. Schematic illustration of photo-current generation in dye solar cells. The photo-current density (J_SC_) can be expressed by $J_{SC}=q \eta_{lh} \eta_{inj} \eta_{cc} \eta_{reg} I_{0}$, where q is the elementary charge (1.6×10^−19^ C), I_0_ is the incident photon flux (m^−2^ s^−1^), η_lh_, η_inj_, η_reg_, and η_cc_ are the efficiencies of light harvesting, photo-excited electron injection, dye regeneration, and charge collection, respectively.

**Figure S11.** Time-resolved PL signals obtained from the MS5 dyes on Al_2_O_3_ film. PL decay curves are compared with and without PEDOT network. Stretched exponential kinetics (ΔOD ∝ $e^{{-t}^{\beta}}$) that are often observed in organic photovoltaic systems is a signature of dispersive environment such that multiple lifetimes are present. That being the case, the simplest approximation for the PL lifetime is to take a decay half time.^[3-4]^

The correlation of PL decay with the interfacial hole transfer is demonstrated by a control experiment where the PL decay of Al_2_O_3_/dye film is observed with and without the HTL. The PL lifetime reduces from 5.2 ns to 3.7 ns after photo-polymerization of PEDOT indicating that hole transfer between the dye and PEDOT causes faster decay dynamics.

**Figure S12**. (A) Concept of photo-induced hole transport study by means of transient photo-voltage. The same cathode material (LFP) is used as the reference. (B) Back-contact potential versus the reference-electrode (E_back vs RE_) is monitored while the pulse LED is controlled. (C) Enlarged photo-voltage rise part.

To confirm the validity of this methodology, we performed a control experiment in which the pulse light intensity is a single variable. The number of photo-injected holes is linearly proportional to the incident light intensity and so is charge reaching the back-contact. As expected, we find that the E_back vs RE_ linearly increases with the intensity of pulse LED (**Figure S12B**), confirming the validity of our method. We also see that the time constant of voltage rise (which is defined as the time taken for E_back vs RE_ to reach 63% of the final value) keeps decreasing with higher LED intensity (**Figure S12C**).

The impedance spectra of PV mode were obtained under dark condition after discharging the battery. The typical impedance spectrum of solid-state dye solar cells is featured by three frequency regimes.^[8, 34]^ The arc at high frequency originates from the back-contact/contacting layer interface (R_back_). In the presented device configuration, it should be dependent on the mixed interface of the back-contact/cathode/HTL. The second arc at intermediate frequency corresponds to the hole transport impedance (R_hole_) while third arc at low frequency represents the charge recombination at the TiO_2_/HTL interface (R_rec_). It is notable that the R_rec_ regime disappears when the device is illuminated at 1-sun condition. This can be explained by higher charge density at the TiO_2_/HTL interface under illumination.^[35]^ Photo-induced charge carriers that are generated at the interface have more chances of recombination. We repeat the impedance analysis (under 1-sun condition) on the PV component of the PRB at different SoC and observe a clear increase of hole transport resistance (R_hole_) as a function of SoC (**Figure 13C**). Hole transport times (τ_hole_) are estimated using the cut-off frequency of the hole transport regime as $\tau_{hole}=1/{2\pi f_{hole}}$. It increases from 61.5 μs to 97.2 μs after full charge as shown in inset of **Figure 13C**, indicating prolonged hole transport kinetics. This result is consistent with the TPV tracking study.

**Figure S13.** Impedance spectra of PV mode measured under dark condition after full discharge: (A) Nyquist plots and (B) Bode plots. One finds the frequency regimes for sequential charge kinetics: interfacial impedance at back-contact/cathode/HTL (ω_back_), hole transport impedance (ω_hole_), and charge recombination at TiO_2_/HTL interface. (C) Impedance spectra measured in PV mode for different SoCs. The measurements are carried out under 1-sun illumination at OCV applied. Inset shows the corresponding Bode plots (θ vs f).

**Figure S14**. (A) Reflectance spectra of cathode layer (LFP@SWCNT@GnP) for optical battery cell and its components (LFP and SWCNT@GnP mixture). (B) Transmittance spectrum of the same cathode layer. (C) *Operando* reflectance spectra obtained from the optical battery cell with different charge voltage. Digital photograph on the right side shows the optical battery cell used. Incorporation of highly conducting SWCNT@GnP composite is key for realizing a current collector-free cathode.

**Figure S15**. Photo-charging current curve of LMO**||**LTO battery depending on the charge mode. Both separate (blue) and integrated (red) charging processes are shown as a function of photo-charged capacity. Time dependent short-circuit currents under PV mode are plotted for comparison (black dashed line).

**References**

[1] N. Schweikert, H. Hahn, S. Indris "Cycling behaviour of Li/Li_4_Ti_5_O_12_ cells studied by electrochemical impedance spectroscopy", *Phys. Chem. Chem. Phys.* **2011**, *13*, 6234.

[2] J. Illig, M. Ender, T. Chrobak, J. P. Schmidt, D. Klotz, E. Ivers-Tiffée "Separation of Charge Transfer and Contact Resistance in LiFePO_4_-Cathodes by Impedance Modeling", *J. Electrochem. Soc.* **2012**, *159*, A952.

[3] S. E. Koops, B. C. O’Regan, P. R. F. Barnes, J. R. Durrant "Parameters Influencing the Efficiency of Electron Injection in Dye-Sensitized Solar Cells", *J. Am. Chem. Soc.* **2009**, *131*, 4808.

[4] J. Guo, J. M. Marin-Beloqui, T. M. Clarke "Bimodal polarons as a function of morphology in high efficiency polymer/acceptor blends for organic photovoltaics", *J. Phys-Mater.* **2021**, *4*, 044009.

[5] B. C. O'Regan, F. Lenzmann "Charge transport and recombination in a nanoscale interpenetrating network of n-type and p-type semiconductors: Transient photocurrent and photovoltage studies of TiO_2_/Dye/CuSCN photovoltaic cells", *J. Phys. Chem. B* **2004**, *108*, 4342.

[6] A. M. Nardes, M. Kemerink, M. De Kok, E. Vinken, K. Maturova, R. Janssen "Conductivity, work function, and environmental stability of PEDOT: PSS thin films treated with sorbitol", *Org. Electron.* **2008**, *9*, 727.

[7] P. P. Prosini, M. Lisi, D. Zane, M. Pasquali "Determination of the chemical diffusion coefficient of lithium in LiFePO_4_", *Solid State Ion.* **2002**, *148*, 45.

[8] A. Dualeh, T. Moehl, M. K. Nazeeruddin, M. Grätzel "Temperature dependence of transport properties of spiro-MeOTAD as a hole transport material in solid-state dye-sensitized solar cells", *ACS Nano* **2013**, *7*, 2292.

[9] N. Li, Y. Wang, D. Tang, H. Zhou "Integrating a photocatalyst into a hybrid lithium–sulfur battery for direct storage of solar energy", *Angew. Chem.* **2015**, *127*, 9403.

[10] M. A. Mahmoudzadeh, A. R. Usgaocar, J. Giorgio, D. L. Officer, G. G. Wallace, J. D. W. Madden "A high energy density solar rechargeable redox battery", *J. Mater. Chem. A* **2016**, *4*, 3446.

[11] S. N. Lou, N. Sharma, D. Goonetilleke, W. H. Saputera, T. M. Leoni, P. Brockbank, S. Lim, D. W. Wang, J. Scott, R. Amal "An operando mechanistic evaluation of a solar‐rechargeable sodium‐ion intercalation battery", *Adv. Energy Mater.* **2017**, *7*, 1700545.

[12] A. Paolella, C. Faure, G. Bertoni, S. Marras, A. Guerfi, A. Darwiche, P. Hovington, B. Commarieu, Z. Wang, M. Prato, M. Colombo, S. Monaco, W. Zhu, Z. Feng, A. Vijh, C. George, G. P. Demopoulos, M. Armand, K. Zaghib "Light-assisted delithiation of lithium iron phosphate nanocrystals towards photo-rechargeable lithium ion batteries", *Nat. Commun.* **2017**, *8*, 14643.

[13] F. Podjaski, J. Kröger, B. V. Lotsch "Toward an aqueous solar battery: direct electrochemical storage of solar energy in carbon nitrides", *Adv. Mater.* **2018**, *30*, 1705477.

[14] Y.-Y. Gui, F.-X. Ai, J.-F. Qian, Y.-L. Cao, G.-R. Li, X.-P. Gao, H.-X. Yang "A solar rechargeable battery based on the sodium ion storage mechanism with Fe_2_(MoO_4_)_3_ microspheres as anode materials", *J. Mater. Chem. A* **2018**, *6*, 10627.

[15] S. Zhang, C. Chen, Y. Zhou, Y. Qian, J. Ye, S. Xiong, Y. Zhao, X. Zhang "TiO_2_-photoanode-assisted direct-solar-energy harvesting and storage in a solar-powered redox cell using halides as active materials", *ACS Appl. Mater. Interfaces* **2018**, *10*, 23048.

[16] B. Lei, G.-R. Li, P. Chen, X.-P. Gao "A quasi-solid-state solar rechargeable battery with polyethylene oxide gel electrolyte", *ACS Appl. Energ. Mater.* **2019**, *2*, 1000.

[17] P. Chen, G. R. Li, T. T. Li, X. P. Gao "Solar‐driven rechargeable lithium–sulfur battery", *Adv. Sci.* **2019**, *6*, 1900620.

[18] B.-M. Kim, M.-H. Lee, V. S. Dilimon, J. S. Kim, J. S. Nam, Y.-G. Cho, H. K. Noh, D.-H. Roh, T.-H. Kwon, H.-K. Song "Indoor-light-energy-harvesting dye-sensitized photo-rechargeable battery", *Energy Environ. Sci.* **2020**, *13*, 1473.

[19] W. Li, J. Zheng, B. Hu, H.-C. Fu, M. Hu, A. Veyssal, Y. Zhao, J.-H. He, T. L. Liu, A. Ho-Baillie "High-performance solar flow battery powered by a perovskite/silicon tandem solar cell", *Nat. Mater.* **2020**, *19*, 1326.

[20] P. Chen, T.-T. Li, Y.-B. Yang, G.-R. Li, X.-P. Gao "Coupling aqueous zinc batteries and perovskite solar cells for simultaneous energy harvest, conversion and storage", *Nat. Commun.* **2022**, *13*, 64.

[21] F. Awai, M. Sasaki, T. Kinoshita, J. Nakazaki, T. Kubo, H. Segawa "Energy-storable dye-sensitized solar cells with improved charge/discharge performance", *Jpn. J. Appl. Phys.* **2023**, *62*, 044001.

[22] L. Jiao, X. Zhang, Y. Feng, J. Lin, D. Yuan, Y. Wang "Coupled solar battery with 6.9% efficiency", *Angew. Chem. Int. Ed.* **2023**, *62*, e202306506.

[23] J. Pan, K. Yuan, X. Mi, Y. Lu, Y. Yu, J. Yang, S. Dou, P. Qin "Efficient bifunctional photoelectric integrated cathode for solar energy conversion and storage", *ACS Nano* **2023**, *17*, 21360.

[24] I. Ciria‐Ramos, E. J. Juarez‐Perez, M. Haro "Solar energy storage using a Cu_2_O‐TiO_2_ photocathode in a lithium battery", *Small* **2023**, *19*, 2301244.

[25] L. Kong, Q. Ruan, J. Qiao, P. Chen, B. Yan, W. He, W. Zhang, C. Jiang, C. Lu, Z. Sun "Realizing Unassisted Photo‐Charging of Zinc–Air Batteries by Anisotropic Charge Separation in Photoelectrodes", *Adv. Mater.* **2023**, *35*, 2304669.

[26] Y. Qu, S. Liao, D. He, L. Duan, J. Wang, D. Yao, M. Ji, F. Lu "Photoreaction Mechanism of the VS_4_-Biomass Carbon Bifunctional Electrode for Photorechargeable Batteries", *ACS Sustain. Chem. Eng.* **2024**, *12*, 4092.

[27] Y. Wang, Y.-T. Chan, T. Oshima, V. Duppel, S. Bette, K. Küster, A. Gouder, C. Scheurer, B. V. Lotsch "Decoupling of Light and Dark Reactions in a 2D Niobium Tungstate for Light-Induced Charge Storage and On-Demand Hydrogen Evolution", *J. Am. Chem. Soc.* **2024**, *146*, 25467.

[28] X.-Y. Du, L.-N. Song, S. Liang, Y.-F. Wang, Y. Wang, H.-F. Wang, J.-J. Xu "Photo-Assisted Chemical Self-Rechargeable Zinc Ion Batteries with High Charging and Discharging Efficiency", *Angew. Chem. Int. Ed.* **2024**, *63*, e202411845.

[29] M. X. Tran, G. Liu, R. E. A. Ardhi, S.-W. Lee, J. K. Lee "A flexible fiber-shaped solar chargeable zinc–polyaniline battery with a fullerene-based electron transfer layer", *Energy Storage Mater.* **2024**, *65*, 103145.

[30] B. Long, X. Liang, Y. Pei, X. Wu, X. Wang, M.-K. Law "Free‐standing BiOI@MWCNTs photoelectrodes for photo‐rechargeable zinc‐ion batteries", *J. Mater. Sci. Technol.* **2024**, *198*, 137.

[31] G. Cheng, Z. Guo, N. Goli, F. Podjaski, K. Zheng, J. Jiang, S. Ramadan, G. Kerherve, S. Tagliaferri, M. Och "Photo-Rechargeable Sodium-Ion Batteries with a Two-Dimensional MoSe_2_ Crystal Cathode", *Nano Lett.* **2025**, *25*, 1775.

[32] B. B. Rath, L. Fuchs, F. Stemmler, A. Rodríguez-Camargo, Y. Wang, M. F. X. Dorfner, J. Olbrich, J. van Slageren, F. Ortmann, B. V. Lotsch "Insights into Decoupled Solar Energy Conversion and Charge Storage in a 2D Covalent Organic Framework for Solar Battery Function", *J. Am. Chem. Soc.* **2025**, *147*, 18492.

[33] X. Zhang, L. Jiao, W. Guo, Z. Xiao, E. Zhou, Y.-X. Tan, Y. Feng, D. Yuan, Y. Wang "A Coupled Organic Solar Battery with 12.1% Efficiency", *J. Am. Chem. Soc.* **2025**, *147*, 21443.

[34] F. Fabregat-Santiago, J. Bisquert, L. Cevey, P. Chen, M. Wang, S. M. Zakeeruddin, M. Gratzel "Electron transport and recombination in solid-state dye solar cell with spiro-OMeTAD as hole conductor", *J. Am. Chem. Soc.* **2009**, *131*, 558.

[35] Q. Wang, J.-E. Moser, M. Grätzel "Electrochemical impedance spectroscopic analysis of dye-sensitized solar cells", *J. Phys. Chem. B* **2005**, *109*, 14945.
